# Accumulation of Seminolipid in Sertoli Cells Is Associated with Increased Levels of Reactive Oxygen Species and Male Subfertility: Studies in Aging *Arsa* Null Male Mice

**DOI:** 10.3390/antiox10060912

**Published:** 2021-06-04

**Authors:** Kessiri Kongmanas, Arpornrad Saewu, Wongsakorn Kiattiburut, Mark A Baker, Kym F Faull, Dylan Burger, Nongnuj Tanphaichitr

**Affiliations:** 1 Chronic Disease Program, Ottawa Hospital Research Institute, Ottawa, ON K1H 8L6, Canada; kessiri.kon@mahidol.ac.th (K.K.); arpornrad@hotmail.com (A.S.); wkiattib@uwo.ca (W.K.); dburger@uottawa.ca (D.B.); 2 Department of Biochemistry, Microbiology, Immunology, Faculty of Medicine, University of Ottawa, Ottawa, ON K1H 8M5, Canada; 3 Division of Dengue Hemorrhagic Fever Research/Siriraj Center of Research Excellence in Dengue and Emerging Pathogens, Faculty of Medicine Siriraj Hospital, Mahidol University, Bangkok 10700, Thailand; 4 Siriraj Metabolomics and Phenomics Center, Faculty of Medicine Siriraj Hospital, Mahidol University, Bangkok 10700, Thailand; 5 Department of Biological Science, University of Newcastle, Callaghan, NSW 2308, Australia; Mark.Baker@newcastle.edu.au; 6 Pasarow Mass Spectrometry Laboratory, University of California, Los Angeles, CA 90024, USA; faull@chem.ucla.edu; 7 Department of Cellular and Molecular Medicine, Faculty of Medicine, University of Ottawa, Ottawa, ON K1H 8M5, Canada; 8 Department of Obstetrics & Gynecology, Faculty of Medicine, University of Ottawa, Ottawa, ON K1H 8L6, Canada

**Keywords:** male fertility, reactive oxygen species, oxidative stress, sperm quality, testicular germ cells, sulfogalactosylglycerolipid, seminolipid, arylsulfatase A, Sertoli cells, storage diseases

## Abstract

Seminolipid (also known as sulfogalactosylglycerolipid-SGG), present selectively in male germ cells, plays important roles in spermatogenesis and sperm–egg interaction. The proper degradation of SGG in apoptotic germ cells is also as important. Sertoli cells first phagocytose apoptotic germ cells, then Sertoli lysosomal arylsulfatase A (ARSA) desulfates SGG, the first step of SGG degradation. We have reported that aging male *Arsa^−/−^* mice become subfertile with SGG accumulation in Sertoli cell lysosomes, typical of a lysosomal storage disorder (LSD). Since reactive oxygen species (ROS) levels are increased in other glycolipid-accumulated LSDs, we quantified ROS in *Arsa^−/−^* Sertoli cells. Our analyses indicated increases in superoxide and H_2_O_2_ in *Arsa^−/−^* Sertoli cells with elevated apoptosis rates, relative to WT counterparts. Excess H_2_O_2_ from *Arsa^−/−^* Sertoli cells could travel into testicular germ cells (TGCs) to induce ROS production. Our results indeed indicated higher superoxide levels in *Arsa^−/−^* TGCs, compared with WT TGCs. Increased ROS levels in *Arsa^−/−^* Sertoli cells and TGCs likely caused the decrease in spermatogenesis and increased the abnormal sperm population in aging *Arsa^−/−^* mice, including the 50% decrease in sperm SGG with egg binding ability. In summary, our study indicated that increased ROS production was the mechanism through which subfertility manifested following SGG accumulation in Sertoli cells.

## 1. Introduction

Sulfogalactosylglycerolipid (SGG) is expressed selectively in mammalian male germ cells at a substantial level [1,2]. SGG is a structural analogue of sulfogalactosylceramide (SGC, also known as sulfatide), present in oligodendrocytes in the brain and epithelial cells of various tissues. Although the two sulfoglycolipids have different backbone neutral lipids, palmitylpalmitoylglycerol (PPG) for SGG and ceramide for SGC, they share the same biosynthesis pathway. Namely, ceramide galactosyltransferase (CGT) galactosylates PPG and ceramide to generate galactosylglycerolipid (GG) and galactosylceramide (GC), which are then sulfated by cerebroside sulfotransferase (CST) to form SGG and SGC, respectively [1]. Mice with global genetic deletion of *Cgt* or *Cst* show tremor and ataxia due to the myelin sheath dysfunction [3,4]. Significantly, *Cgt^−/−^* and *Cst^−/−^* male mice are infertile [4,5]. In WT mice, SGG is first synthesized in primary spermatocytes [1], and in these two knockout (KO) mice, spermatogenesis is halted at this germ cell stage [4,5]. The results indicate that SGG is indispensable in this process. Furthermore, our accumulated studies reveal the significance of sperm SGG in fertilization [6,7]. SGG is also a critical component in the formation of sperm lipid raft membranes [8,9], which are the interaction platforms between sperm and the egg extracellular matrix, the zona pellucida (ZP) [8,10]. Additionally, SGG possesses inherent affinity to the ZP [6,8]. 

SGG also shares the same degradation pathway with SGC, involving two lysosomal enzymes (and their co-enzymes), i.e., arylsulfatase A (ARSA) (and saposin B) to produce GG and GC, and galactosyl ceramidase (GALC) (and saposin A) to generate their corresponding backbone neutral lipids, PPG and ceramide, respectively [1]. This degradation pathway was first discovered for SGC due to the natural mutations in humans of *ARSA* and *GALC,* which lead to the lysosomal accumulation of SGC and GC, respectively, and subsequent lysosomal distension, as well as cytotoxicity. These lysosomal storage disorders (LSDs) are termed metachromatic leukodystrophy (MLD) and globoid cell leukodystrophy (also known as Krabbe disease) for the deficiency of ARSA and GALC activities, respectively. Individuals with MLD or Krabbe disease suffer severe neurological and motor neuron disorders caused by the demyelination of their neuron cells, and they usually die young [11,12]. Similarly, *Galc* mutant mice (also known as twitcher *Twi*/*Twi*) exhibit body tremors, and for most strains, they die young. Only one twitcher mouse strain can live up to 50 days [13], slightly beyond the first spermatogenesis wave. In these twitcher mice, GG is accumulated in Sertoli cells, implicating the role of GALC in degalactosylating GG to PPG. These twitcher mice are infertile, owing to the production of abnormal sperm and architectural impairment of the epididymis [14,15]. 

Mice with global genetic deletion of *Arsa* were generated for studies of MLD molecular mechanisms [16]. *Arsa* null mice exhibit SGC accumulation in the brain as expected [1]. However, they have mild symptoms of MLD [16] and they can live at least to 18 months of age (our unpublished observation). We have previously demonstrated by mass spectrometry-based lipidomic analyses that the levels of SGG are increased in the testis of *Arsa* null mice, as compared with the levels in age-matched wild types. Immunofluorescence studies indicate that this increase is likely attributed to SGG accumulation in *Arsa* null Sertoli cells. Electron microscopy further reveals that lysosomes in Sertoli cells of aging *Arsa*^−/−^ mice are much distended, with numerous lipid droplets present in these cells, all characteristics typical of an LSD [17]. Aging *Arsa*^−/−^ mice (more than 5 months of age) are subfertile with significant issues in spermatogenesis, an increase in apoptotic TGCs in the seminiferous tubules, abnormal sloughing of TGCs into the seminiferous tubule and epididymal lumen, abnormal morphology of sperm produced and a minimal ability of epididymal sperm to fertilize eggs in vitro [17]. Since Sertoli cells are sustentacular cells, with essential roles in supporting spermatogenesis [1], the abnormalities in the spermatogenesis events mentioned above are likely the consequences of dysfunctionality of Sertoli cells in *Arsa*^−/−^ mice. The primary objective of this report was to discern the molecular mechanisms, which caused these dysfunctions. Based on previous reports in a number of LSDs that oxidative stress is the mechanism of LSD-induced cytotoxicity [18], our study was targeted towards measuring ROS in Sertoli and TGCs of *Arsa*^−/−^ mice. With the recently available method to isolate Sertoli cells from adult mice with high purity [19], we also confirmed the accumulation of SGG in these cells from *Arsa*^−/−^ mice by quantitative electrospray ionization-tandem mass spectrometry (ESI-MS/MS). 

## 2. Materials and Methods

### 2.1. Mice

*Arsa^−/−^* mice originally created by Hess et al. [16] were rederived by Dr. Tony Rupar at the Animal Care and Veterinary Services, University of Western Ontario, London, Ontario, Canada by breeding them into the C57BL/6 background. A pair of male and female *Arsa^−/−^* mice and wild type C57BL/6 mice were generously provided to us. Breeding between *Arsa^−/−^* males and *Arsa^−/−^* females was performed to maintain the colony and to supply *Arsa^−/−^* males for this study. Wild type C57BL/6 males were either obtained from in-house breeding or purchased from Charles River (Senneville, QC, Canada). All mice were kept in a temperature-controlled room with 14-h light and 10-h dark photoperiod and were fed ad libitum with Purina rodent chow and water. The use of mice (protocol 2568) was approved by the University of Ottawa Animal Care Committee, which endorses the use of ARRIVE checklists and guideline.

### 2.2. Isolation of Tissues and Cells

Testes and caput and cauda epididymides were removed free from the surrounding fat pad and/or blood vessels from male mice sacrificed by cervical dislocation. Caput and cauda epididymides were used directly for incubation with dihydroethidium (DHE, see below). In another set of experiments, sperm were collected from the cauda epididymis for further processing (see below). 

#### 2.2.1. Seminiferous Tubules and Sertoli cells

To prepare seminiferous tubules, decapsulated testes were subjected to collagenase digestion. Details of enzymatic digestions were as previously described [19]. The seminiferous tubules obtained after collagenase digestion were either used directly for DHE incubation (see below) or further subjected to a series of enzyme digestions [19], which finally yielded loose cells comprising Sertoli cells, TGCs, heads and tails of elongated spermatids and testicular sperm as well as tubule remnants (incompletely digested by the enzymes). A primary culture of Sertoli cells was then prepared from this cell mixture according to our described method [19]. 

#### 2.2.2. Testicular Germ Cells

TGCs were prepared by incubating (15 min, 35 °C) seminiferous tubules (isolated as described above from two testes) with 3 mL of 0.25 mg/mL trypsin and 10 μg/mL DNase I in DMEM/F12 (made without phenol red) in a shaking (150 rpm) water bath. During the last 3 min, the suspension was gently pipetted up and down with a transfer pipet to accelerate the dispersion of the tubules. After stopping the trypsin reaction with 0.25 mg/mL soybean trypsin inhibitor (STI), the suspension was centrifuged (800× *g*, 3 min, room temperature (RT)) to pellet the dispersed cells and undigested tubule fragments, which were washed once by centrifugation in DMEM/F12. The suspension was then incubated (20 min, 35 °C) with 3 mL of 1 mg/mL collagenase, 2 mg/mL hyaluronidase, 10 μg/mL DNase I, 0.25 mg/mL STI in DMEM/F12 in the shaking water bath to further dissociate the remaining undigested tubules. At the end of the incubation, the dissociated cells and tubule remnants were washed twice by centrifugation (800× *g*, 3 min, 28 °C) with DMEM/F12 or another buffer solution specific for the downstream experiment. The tubule remnants were then removed by passing the cell suspension through a 100 μm cell strainer, and the filtrate containing loose cells was subjected to cell counting. The majority (95–97%) of intact cells in the filtrate were TGCs, with only 3–5% being Sertoli cells. This cell suspension was therefore used to represent TGCs in immunoblotting and DHE experiments (see below).

### 2.3. Collection of Caudal Epididymal and Vas Deferens Sperm and Their Separation into Percoll Gradient-Interfaced and Percoll-Gradient-Pelleted Populations 

Sperm were collected as milk-like fluids from the cauda epididymis and vas deferens into KRB-HEPES as previously described [20]. The sperm fluid collected from either 5 or 8-month old WT and *Arsa* null mice were subjected to Percoll gradient centrifugation (1 mouse/gradient for both ages of WT mice and 5-month-old *Arsa* null mice; 2–3 mice/gradient for 8-month-old *Arsa* null mice). The sperm fluid was resuspended in 2 mL of KRB-HEPES and then subjected to centrifugation (650× *g*, 30 min, 28 °C) through a Percoll gradient (35% and 70% solution made in KRB-HEPES, 2 mL each), which separated sperm into the Percoll-gradient interfaced and Percoll-gradient pelleted populations. These two sperm populations were washed once in KRB-HEPES by centrifugation (500× *g*, 5 min, 28 °C) and then applied onto a slide for staining with Diff-Quik solution (Siemens Healthcare Limited, Oakville, ON, Canada) following the manufacturer’s instruction. The morphology of stained sperm was viewed under a Zeiss Axioscope microscope. Percoll-gradient centrifuged (PGC) sperm (the pellet fraction) were also used for sulfolipid quantification (see below).

### 2.4. Immunofluorescence of Sertoli Cells for ARSA, LAMP1, Saposin B, and SGG

Primary cultures of Sertoli cells in an ibidi high-walled μ-Dish 35-mm plate dish (ibidi USA, Inc., Fitchburg, WI, USA) with at least 70% confluence were used for immunofluorescence to co-localize ARSA, saposin B or SGG with LAMP1. Primary antibodies and corresponding secondary antibodies are as described in Supplemental Table S1. Sertoli cell cultures were first washed with PBS, which was used for washing the cultures in between all treatment steps and also for the dilution of all reagents. They were then fixed (15 min, RT) with 4% paraformaldehyde (PFA) and permeabilized (5 min, RT) with 0.1% Triton X-100. Non-specific binding was blocked (1 h, RT) using the blocking solution specific for each secondary antibody (Supplemental Table S1). The culture was subsequently incubated (1 h, RT) with the primary antibody and the corresponding secondary antibody (1 h, RT). In all co-localization pairs, the incubation with anti-LAMP1 and its secondary antibody was performed after the incubation with anti-ARSA, anti-saposin B or anti-SGG and its secondary antibody. Sertoli cell cultures incubated with isotype IgG or IgM control, in place of the primary antibody, served as negative controls. After completing immunofluorescence steps, the culture was incubated with Hoechst 33258 (5 μg/mL, Molecular probes: cat. no. H-2491). The Sertoli cell cultures were then viewed under a Zeiss Axio epifluorescence microscope using the rhodamine filter for LAMP1 detection, the fluorescein filter for other antigens and the Hoechst filter for the Hoechst dye. Images of the three filters were merged for presentation.

### 2.5. Immunoblotting for ARSA

TGCs and Sertoli cells detached from the substratum by treatment with Accutase Cell Detachment Solution (BD Biosciences) were treated (90 °C, 5 min) with 2% SDS in 0.0625 M Tris-HCl, pH 6.8. At the end of the treatment, particulates were removed from the treated cells by centrifugation (14,000× *g*, 5 min, RT). Proteins in the supernatant were then quantified using the BCA Protein Assay Kit (Thermo Fisher Scientific, Waltham, MA, USA) and added with DTT to the final concentration of 100 mM. Proteins (50 μg) were subjected to Laemmli SDS-PAGE (on a 10% polyacrylamide gel) and the electrophoresed proteins were electro-transferred (100 V, 1 h) [21] onto a nitrocellulose membrane (Bio-Rad Laboratories) for immunoblotting with anti-ARSA. The membrane was first blocked (1 h, RT) for non-specific binding in 5% nonfat skim milk in TBST (10 mM Tris-HCl, pH 7.4, 150 mM NaCl, 0.1% Tween 20) and then incubated (12 h, 4 °C) with anti-ARSA (MyBiosource, 1:1000 dilution in the blocking solution). This was followed by incubation (1 h, RT) with the secondary antibody—HRP-conjugated rabbit anti-goat IgG antibody (Bio-Rad, 1:3000 dilution in the blocking solution). In between incubation steps, the membrane was washed twice with TBST (10 min per wash, RT). The antigen–antibody complex was revealed by enhanced chemiluminescence on a GE Healthcare Amersham Hyperfilm (Thermo Fisher Scientific) using a SuperSignal West Femto Maximum Sensitivity substrate (Thermo Fisher Scientific).

### 2.6. Lipidomic Analyses of Sertoli Cells and Sperm 

Using a modified Bligh and Dyer method [2,22], lipids were isolated from Sertoli cells detached from the culture plate by Accutase treatment. Major lipids from the same numbers of WT and *Arsa^−/−^* Sertoli cells were then comparatively quantified by different modes of flow injection/ESI-MS/MS coupled with multiple reaction monitoring (MRM) as previously described [1,19,23]. Lipids extracted from 0.04 million and 0.008 million Sertoli cells were used per injection for initial global analyses and subsequent MRM quantifications, respectively. Lipids analyzed included sulfolipids (SGG and cholesterol sulfate (CS)), identified by precursor ion scanning in the negative mode (parents of *m*/*z* 97 (sulfate group)); phosphatidylcholines (PCs) and sphingomyelins (SMs), identified by precursor ion scanning in the positive mode (parents of *m*/*z* 184 (choline group)); phosphatidylethanolamines (PEs), identified by neutral loss scanning in the positive mode (loss of *m*/*z* 141 (ethanolamine group)); cholesteryl esters (CEs), identified by precursor ion scanning in the positive mode (parents of *m*/*z* 364.9 (cholesteryl group)). The identities of candidate lipids were assigned by manually comparing the *m*/*z* values obtained from the MS/MS analyses with those listed in the online lipid databases (retrieved on 15 December 2018 from http://www.lipidmaps.org and http://www.byrdwell.com/LipidAcademy). For SGG, its absolute amounts in Sertoli cells of WT and *Arsa* null mice were further obtained through flow injection/ESI-MS/MS-MRM by comparing the peak areas of parents of *m*/*z* 97 in the samples with those of a standard curve built with known amounts (0.2–20 pmole/10-μL injection) of the deuterated form of SGG synthesized by us [24]. Since sulfatide (C16:0 SGC), upon subjection to ESI-MS/MS coupled with multiple reaction monitoring (MRM), produced the sulfate *m*/*z* 97 ion like SGG and deuterated SGG, SGC was quantified from the *m*/*z* 97 ion standard curve generated from the deuterated SGG standard. For lipids other than SGG and SGC, relative abundance between those present in WT and *Arsa^−/−^* Sertoli cells was compared based on peak areas of each molecular species.

Lipids were also extracted from PGC sperm of 8-month-old WT and *Arsa^−/−^* males and quantified for SGG and cholesterol sulfate (the two major sperm sulfolipids) by ESI-MS/MS-MRM using deuterated SGG and cholesterol sulfate to build standard curves in a similar manner as described above. 

Total cholesterol (free and CEs) in lipids extracted from Sertoli cells was quantified using Molecular Probes Amplex Red Cholesterol Assay Kit (Thermo Fisher Scientific, A12216) following the manufacturer’s instruction. CEs present in the lipid sample were hydrolyzed by cholesterol esterase (provided in the kit) to cholesterol. In this assay, all free cholesterol was oxidized by cholesterol oxidase (provided in the kit) to yield H_2_O_2_ and cholestene-3-one. The produced H_2_O_2_ then reacted with the provided Amplex Red reagent, 10-acetyl-3,7-dihydroxyphenoxazine, as catalyzed by HRP (provided in the kit) to generate highly fluorescent resorufin. This assay was performed in a black-walled 96-well plate (Corning Inc., Corning, NY, USA). In each well of the plate, the lipid extract from 0.01 million Sertoli cells dissolved in 25 μL of 100% ethanol was mixed with 25 μL of 1X Reaction Buffer (100 mM potassium phosphate, pH 7.4, 50 mM NaCl, 5 mM cholic acid and 0.1% Triton X-100). This lipid solution was incubated (30 min, 37 °C) in the dark with 50 μL of the Working Solution (0.2 U/mL cholesterol esterase, 2 U/mL HRP, 2 U/mL cholesterol oxidase, 300 μM of Amplex Red reagent in 1X Reaction Buffer). In order to construct the cholesterol standard curve, cholesterol (0 to 1 μg) (in place of Sertoli cell lipids) was also subjected to the same incubations. The fluorescence of resorufin generated was measured in a fluorescence microplate reader, SpectraMAX GeminiXS (Molecular Devices, Sunnyvale, CA, USA), using the excitation and emission wavelengths of 560 and 590 nm, respectively. Background fluorescence was measured from the well that contained the same volumes of all solutions but without any lipids and the value was used for subtraction from each data value. The assay was performed in duplicates of each Sertoli cell lipid sample. 

### 2.7. Measurement of Superoxide Anion Levels in Sertoli Cells, Testicular Germ Cells, Seminiferous Tubules and Caput and Caudal Epididymis 

#### 2.7.1. Sertoli Cells

Sertoli cells cultured to ~70–80% confluence on culture day 7 in a 6-cm plate were washed twice with PBS containing 100 μM of diethylenetriaminepentaacetic acid (DTPA, Sigma) (PBS/DTPA, 3 mL each wash). The Sertoli cell culture was then incubated (30 min, 35 °C, 5% CO_2_) with 3 mL of Gibco HBSS (Thermo Fisher Scientific, A14026-076) containing 100 μM of DTPA (HBSS/DTPA) and 30 μM of DHE (Sigma). At the end of the incubation, the Sertoli cell culture was washed twice with PBS/DTPA (3 mL each wash) and the cells were detached from the plate by treatment (5 min, 35 °C, 5% CO_2_) with 3 mL of Accutase. Sertoli cells were collected in a 15-mL tube and washed twice in PBS-DTPA (3 mL each wash) by centrifugation (800× *g*, 3 min, 28 °C). The Sertoli cell pellet was added with 1 mL of acetonitrile and the mixture was sonicated in a cup using a Fisher Sonic Dismembranator Model 300 at 90% output for 3 min. Following centrifugation at 12,000× *g* for 10 min at 4 °C to pellet cell particulates, the cell extract in the supernatant was transferred to a fresh tube and dried under vacuum. The dried cell extract was kept at −80 °C in the dark until HPLC analyses. 

#### 2.7.2. Testicular Germ cells

TGCs (~1 million) prepared as described above were incubated (15 min, 35 °C, 5% CO_2_) in 500 μL of HBSS/DTPA containing 1 μM of PMA (phorbol myristate acetate (Sigma)), diluted from the 16 mM stock solution, which was made in DMSO). The cells were then washed twice in 500 μL of HBSS/DTPA (800× *g*, 3 min, RT) and incubated (10 min, 35 °C, 5% CO_2_) in 500 μL of HBSS/DTPA containing 200 μM of DHE. The unincorporated DHE was then washed off twice from TGCs with 500 μL of PBS/DTPA by centrifugation (1000× *g*, 5 min, 4 °C). Acetonitrile (500 μL) was added to the TGC pellet and the mixture was sonicated at 90% output for 3 min. The particulates were then removed by centrifugation and the cell extract was dried under vacuum and kept frozen in the same manner as described for Sertoli cells. 

#### 2.7.3. Seminiferous Tubules

The tubules isolated from two decapsulated testes as described above were washed twice in 5 mL of DMEM/F12 (containing no phenol red). They were allowed to sediment by unit gravity during the washes and were finally separated from the supernatant by passing the suspension through a 100 μm cell strainer. The tubules were retained on the strainer and a small fraction of them were taken with the best attempt to remove residual medium from them. These tubules were about 1 mg (ranging from 0.9 to 1.1 mg), as revealed by weighing. They were placed in a 1.5 mL Eppendorf tube containing 250 μL of PBS/DTPA and cut into small (1–2 mm) pieces using small scissors. These tubule pieces were washed twice in the same buffer by centrifugation (100× *g*, 3 min, 28 °C), and then incubated (15 min, 35 °C) in 500 μL of PBS/DTPA containing 200 μM of DHE. At the end of the incubation, the tubules were washed (100 g, 3 min, RT) twice in 500 μL of PBS/DTPA and the tubules sedimented as a pellet were snapped frozen in liquid nitrogen. The frozen tubule pellet was homogenized using a micro homogenizer and added with 500 μL of acetonitrile. This was followed by sonification at 90% output for 3 min. The sonicated tissue suspension was centrifuged at 12,000× *g* for 10 min at 4 °C to pellet the particulates. The tissue extract in the supernatant was then vacuum dried and kept frozen until HPLC analyses. 

#### 2.7.4. Caput and Cauda Epididymides

A caput and cauda epididymis were dissected from a WT or *Arsa* null mouse and immediately cut into 1–5 mm pieces. Similar to the procedures used for seminiferous tubules, the epididymis pieces (~1 mg) were washed in PBS/DTPA, incubated in HBSS/DTPA containing DHE, and processed to obtain the cell extract, as described above. 

#### 2.7.5. Quantification of Superoxide Anion

Incorporated DHE was converted specifically by the superoxide anion through one-electron oxidation to 2-hydroxyethidium (2HE). However, DHE was also oxidized through a two-electron reaction by hydrogen peroxide and other oxidants inside the cells to become ethidium (Eth), which has a similar excitation and emission spectra as 2HE. Therefore, 2HE and Eth, as well as unreacted DHE, were first separated from each other by HPLC [25,26]. The dried samples were each dissolved in 120 μL of 20% methanol and 0.1% trifluoroacetic acid and 100 μL of the suspension was injected into an Agilent 1100 HPLC system with an Agilent Zorbax 300SG C18 column (5 μm internal diameter). A gradient consisting of solution A (20% methanol and 0.1% trifluoroacetic acid) and solution B (100% methanol) was applied to the column to separate DHE, Eth and 2HE from each other. The gradient started at 0% solution B and was increased linearly to 50% solution A within the first 10 min. Solutions were then held for 10 min followed by a wash with 100% solution B for 5 min. The system was subsequently equilibrated with 100% solution A for a further 10 min. The DHE peak was detected by its UV absorption at 245 nm, whereas detection of the Eth and 2HE was carried out by their fluorescence signals with the excitation and emission wavelengths set up at 480 and 580 nm, respectively. All separated products were quantified by comparing the peaks to those of the known standards and the results were expressed as the ratio of 2HE (nmole) to DHE (μmole) and Eth (nmole) to DHE (μmole). The former ratio 2HE/DHE specifically reflected the amount of superoxide anion inside the cells/tissues. Both the 2HE/DHE and E/DHE in cells and tissues of *Arsa^−/−^* mice were expressed as relative to the corresponding values in age-matched WT mice, which were designated as 1. 

### 2.8. Measurement of Hydrogen Peroxide in Sertoli Cells and Epididymal Fluid

H_2_O_2_ produced by Sertoli cells or present in epididymal fluid was quantified using Molecular Probes Amplex Red Hydrogen Peroxide Assay Kit (Thermo Fisher Scientific, A22188) with a modification. In this assay, HRP (provided in the kit) catalyzes the oxidation by H_2_O_2_ and other oxidants in the cells of the Amplex Red reagent into fluorescent resorufin. 

Sertoli cells cultured in two 10-cm plates to ~70–80% confluence on culture day 7 were first washed twice with sterile PBS (prewarmed to 35 °C) (5 mL/plate/wash). Each Sertoli cell plate was then incubated with 3 mL of 1X Reaction Buffer (0.05 M sodium phosphate, pH 7.4) at 35 °C for 1 h. At the end of the incubation, the cells were scraped off both plates and combined for centrifugation (500× *g*, 3 min). The supernatant was then discarded and the cell pellet was resuspended in 1X Reaction Buffer, so that the final volume of the cell suspension was 220 μL. For each assay point, 50 µL of the Sertoli cell suspension was used and the assay of each sample was performed in triplicates in a black-walled 96-well plate. Another 50 µL of the same Sertoli cell suspension was also placed in a well of the 96-well plate for treatment (1 h, 37 °C) with 10 µL of polyethylene glycol (PEG)-catalase (500 IU, Sigma, cat. no. C4963; stock solution made in 1X Reaction Buffer), which would induce the breakdown of H_2_O_2_ into H_2_O and O_2_. The triplicate wells of untreated Sertoli cell suspension were added with 10 µL of Reaction Buffer and incubated in parallel. Finally, all wells were added with 50 µL of the Working Solution containing 100 µM Amplex Red reagent and 0.2 U/mL HRP in the Reaction Buffer and incubated (30 min, RT) in the dark. Fluorescent resorufin generated was then measured using a FLUOstar Galaxy Microplate Reader (BMG Labtechnologies, Ortenberg, Germany) with the excitation and emission setups as described in the cholesterol assay. The fluorescence specific to H_2_O_2_ induction was obtained by subtracting the untreated sample value with the catalase-treated value.

An aliquot (10 μL) of the remaining Sertoli cell suspension was subjected to cell counting using a hemocytometer. The fluorescent values or resorufin obtained were then expressed as arbitrary fluorescence units per 0.2 millions of Sertoli cells.

H_2_O_2_ in the epididymal fluid was also quantified. Using a pair of forceps, epididymal fluid containing sperm was squeezed out from the cauda epididymis pre-scored longitudinally with a razor blade. The milk-like fluid was immersed immediately into 200 μL of KRB-HEPES, which was subjected to centrifugation (1000× *g*, 10 min, 28 °C) to pellet sperm. The supernatant was re-centrifuged at 14,000× *g* (5 min, 28 °C) to pellet any cellular debris and particulates. The final supernatant representing diluted epididymal fluid (50 μL) was used for H_2_O_2_ quantification, following the same protocol as described above. The resorufin fluorescent values were expressed as arbitrary fluorescence units per 10 μL of epididymal fluid.

### 2.9. Measurement of Antioxidants in Epididymal Fluid 

Total antioxidant levels in epididymal fluid of 8-month-old *Arsa^−/−^* and WT mice were determined using the Antioxidant Assay Kit (Cayman Chemical, MI, USA, Cat. No. 709001). This assay was based on the ability of antioxidants in the sample to inhibit the oxidation of ABTS (2,2′-azino-di-[3-ethylbenzthiazoline sulphonate]) to ABTS•+ (with absorbance at 750 nm) by metmyoglobin. In parallel, the assay was set up with a positive control antioxidant, Trolox (a water-soluble tocopherol analogue), in place of the sample. The levels of antioxidants in the sample were quantified as millimolar Trolox equivalents. 

To prepare the sample from each mouse, the epididymal fluid containing sperm was collected from the cauda epididymis into 1 mL of PBS. This was followed by the removal of sperm and cellular debris and particulates by centrifugation as described above. The supernatant was then diluted 1:10 with the Assay buffer (5 mM potassium phosphate, pH 7.4, containing 0.9% sodium chloride and 0.1% glucose) prior to assaying following the manufacturer’s instruction. The antioxidant concentrations were reported as millimolar Trolox equivalents in the original epididymal fluid collected in 1 mL of PBS. 

### 2.10. Detection of Apoptosis in Sertoli Cells

The late stage of apoptosis in Sertoli cells was detected by the presence of nicks in DNA, using a Roche In Situ Cell Death Detection Kit, TMR Red (Millipore Sigma), following the manufacturer’s instructions. Sertoli cells were prepared as described above with the exception that DNase I was omitted in all enzyme solutions. DMEM/F12 with proper supplements were used in the cell preparation and culture prior to the apoptosis assay. Approximately, 1–2 × 10^6^ Sertoli cells, Accutase detached from the substratum, were washed (800× *g*, 3 min, RT) once in 100 μL of PBS and then fixed (1 h, RT) in 100 μL of 4% PFA. The cells were then washed (800× *g*, 3 min, RT) in PBS twice and the cell suspension was divided equally into three aliquots. One aliquot serving as a negative control was incubated (60 min, 37 °C) in the dark with 50 μL of Label Solution (containing only tetramethylrhodamine (TMR)-dUTP, provided in the kit). The second aliquot to be assessed for the presence of nicks in DNA in vivo was incubated (60 min, 37 °C) in the dark with 50 μL of TUNEL Reaction Mixture (prepared by mixing Label Solution and Enzyme Solution (containing deoxynucleotidyl transferase (TdT) enzyme) as per instruction in the kit). The third aliquot serving as a positive control was treated (15 min, RT) with DNase I (200 μg/mL) in 50 mM Tris-HCl containing 0.1% BSA to generate as many nicks as possible in DNA. After the treatment, cells in the third aliquot were incubated with TUNEL Reaction Mixture in the same manner as cells in the second aliquot. Cells of the three aliquots were then washed (800 g, 3 min, RT in 100 μL of PBS) twice, resuspended in 30 μL of PBS and applied onto a slide topped with a cover slip for viewing under a Zeiss Axio epifluorescence microscope using the rhodamine filter.

### 2.11. Statistical Analyses

All quantitative results were expressed as means ± SDs from 3 or more replicate samples. Student’s *t*-test was used to determine significant differences of the data between *Arsa^−/−^* and WT samples.

## 3. Results

### 3.1. Localization of ARSA, Saposin B and SGG in the Lysosomes of WT Sertoli Cells

**Figure 1:**
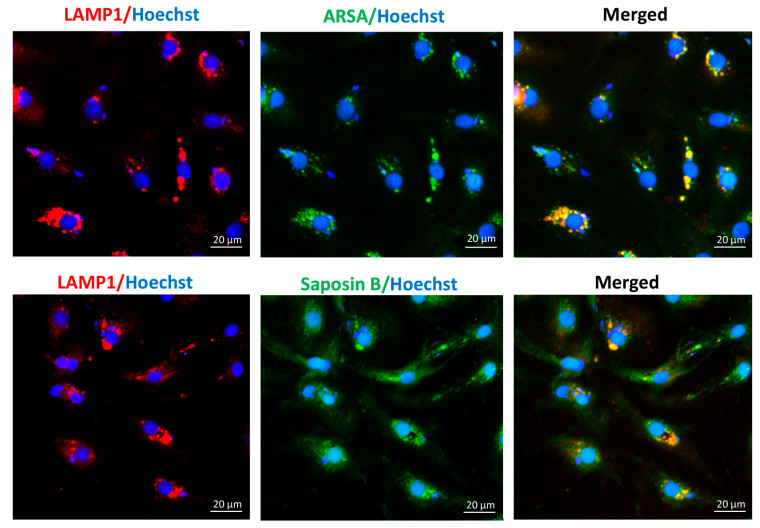
Immunolocalization of ARSA and LAMP1 (**top panel**), and saposin B and LAMP1 (**bottom panel**) in Sertoli cells from 8-month-old WT mice. Left and middle panels show LAMP1 and ARSA or saposin B localization, respectively, whereas the right panels display the merged signals of ARSA with LAMP1 and saposin B with LAMP1. Results shown are representative from three replicate experiments.

**Figure 2:**
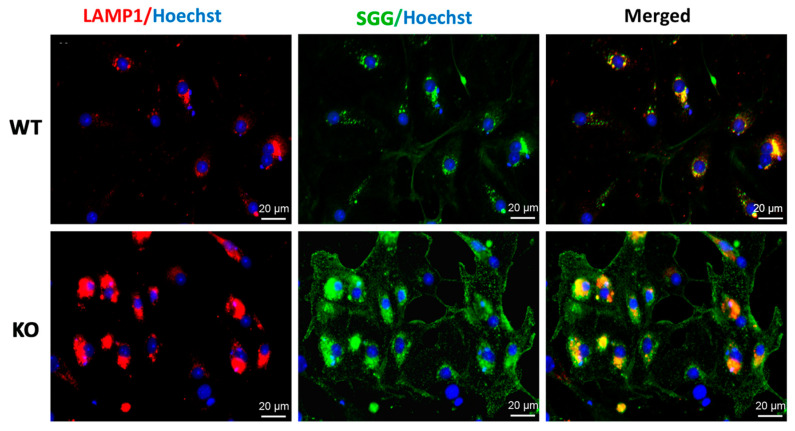
Immunolocalization of SGG and LAMP1 in Sertoli cells from 8-month-old WT mice (**top panel**) and in Sertoli cells from age-matched *Arsa^−/−^* mice (**bottom panel**). Left and middle panels show LAMP1 and SGG localization, respectively, whereas the right panels display the merged signals of SGG with LAMP1. Results shown are representative from three replicate experiments.

Immunofluorescence studies revealed that ARSA, saposin B, and the majority of SGG were co-localized with LAMP1 (a lysosome marker), in primary cultures of Sertoli cells from 8-month-old WT mice (Figure 1 and Figure 2), indicating that these macromolecules were present in the lysosome. This was expected, since the enzyme ARSA and its co-enzyme, saposin B, have been known as lysosomal components with SGG as their substrate [1,27]. However, anti-saposin B-reacting signals were also observed in the cytoplasm and may have reflected the precursor form of saposin B, i.e., prosaposin, which is known for its presence in the Sertoli cell cytoplasm [28]. On the other hand, a small population of SGG in the cytoplasm with a granular pattern was noted (Figure 2), suggesting its presence in membranated organelles such as endosomes. It was possible that this SGG population was the newly phagocytosed lipid, which had not yet been transported to lysosomes.

### 3.2. Increased Amounts of SGG and Its Additional Localization in the Cytoplasm in Sertoli Cells of 8-Month-Old Arsa Null Mice 

#### 3.2.1. Immunofluorescence Studies

Increases in SGG amounts in Sertoli cells from 8-month-old *Arsa* null mice, as compared with Sertoli cells from age-matched WTs, were observed. These increases were evident by higher immunofluorescence intensity of SGG in the lysosome as well as strong immunofluorescent signals present as the granular pattern in the cytoplasm as well as on the cell surface of *Arsa*^−/−^ Sertoli cells (Figure 2, bottom panels vs. top panels). The results indicated that SGG was accumulated in *Arsa*^−/−^ Sertoli cells due to the failure to be catabolically processed because of the lack of ARSA (see Supplemental Figure S1), the first enzyme in the SGG degradation pathway [1]. However, saposin B was still present in the lysosome and cytoplasm of *Arsa*^−/−^ Sertoli cells, similar to the observation in the WT counterparts (Supplemental Figure S2). It was also noted that the LAMP1 signal in *Arsa*^−/−^ Sertoli cells was much stronger than that in WT Sertoli cells (Figure 2), implicating the distention of the organelle.

#### 3.2.2. Lipidomic Analyses of SGG and Other Lipids

**Figure 3:**
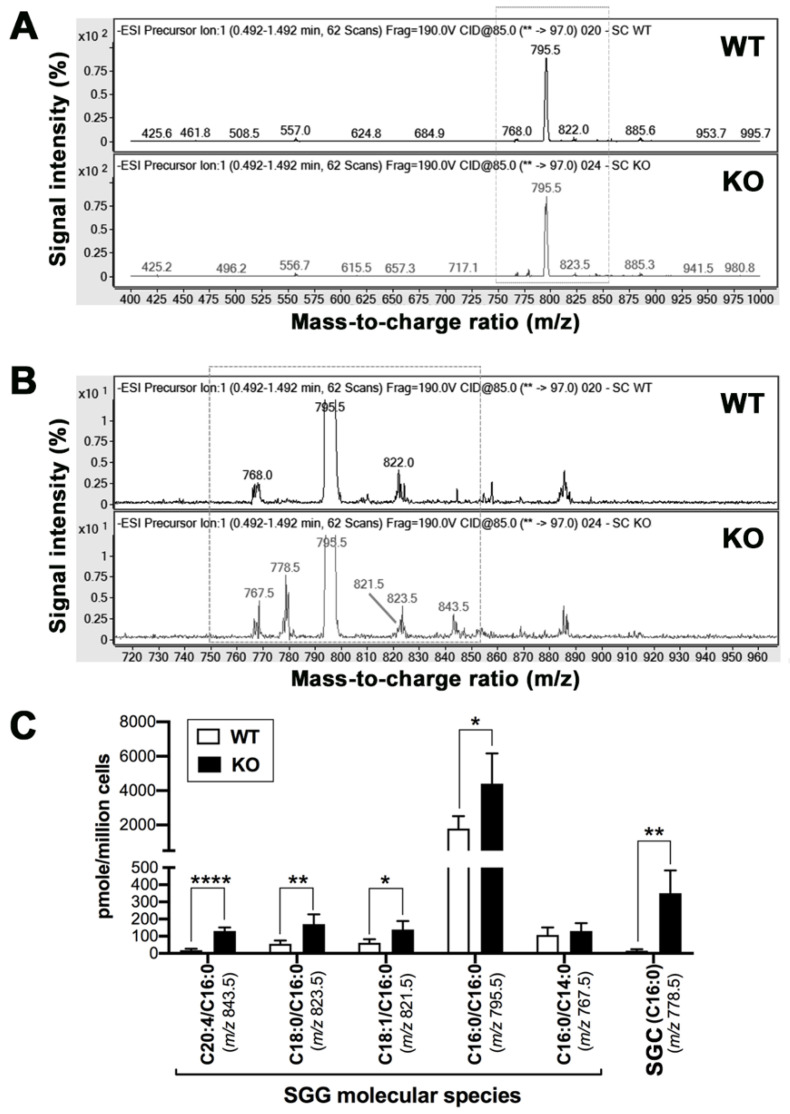
ESI/MS-MS spectra from precursor ion scanning of *m*/*z* 97 (sulfate group) showing major sulfolipids in Sertoli cells from 8-month-old wild type (WT) and *Arsa* KO mice. (**A**) Representative mass spectra of one out of four sets of WT and KO samples were shown. Note that SGG (C16:0/C16:0) with *m*/*z* 795.5 was the most abundant sulfolipid in both WT and KO Sertoli cells. (**B**) Magnified view of the mass spectra in the range of *m*/*z* 720-960 showing the presence of three SGG molecular species (C16:0/C14:0 with *m*/*z* 768.0/767.5; C18:1/C16:0 with *m*/*z* 821.5/822.0; C18:0/C16:0 with *m*/*z* 823.5) in both WT and KO Sertoli cells, as well as the selective presence of another SGG molecular species, (C20:4/C16:0, *m*/*z* 843.5), and a sulfatide SGC (C16:0) with *m*/*z* 778.5 in the KO sample. (**C**) Comparative amounts of various SGG molecular species and sulfatide (SGC (C16:0)), as determined by tandem MS coupled with MRM, in WT and *Arsa* KO Sertoli cells. Results shown are from four replicate experiments. *, **, **** denote *p* < 0.05, 0.01 and 0.0001, respectively.

ESI-MS/MS revealed that SGG was the prevalent sulfolipid present in Sertoli cells of both 8-month-old WT and *Arsa* null mice, with the SGG (C16:0/C16:0, *m*/*z* 795.5) being the major molecular species (Figure 3A). However, low amounts of other SGG molecular species, including the SGG (C16:0/C14:0, *m*/*z* 768.0/767.5), (C18:1/C16:0, *m*/*z* 822.0/821.5) and (C18:0/C16:0, *m*/*z* 823.5) species were also present in WT and *Arsa^−/−^* Sertoli cells. On the other hand, an additional SGG molecular species (C20:4/C16:0, *m*/*z* 843.5) and an analogue of SGG, sulfatide SGC (C16:0, *m*/*z* 778.5) were selectively present in *Arsa^−/−^* Sertoli cells (Figure 3B,C). Quantitative analyses by MRM indicated that the amount of SGG (C16:0/C16:0) in *Arsa^−/−^* Sertoli cells was twice that of the WT counterpart (4.40 ± 1.76 versus 1.79 ± 0.73 nmole/10^6^ cells with *p* < 0.05). The levels of the SGG molecular species (*m*/*z* 843.5, 821.5 and 823.5) and SGC (*m*/*z* 778.5) were also significantly higher in *Arsa^−/−^* Sertoli cells (Figure 3C).

**Figure 4:**
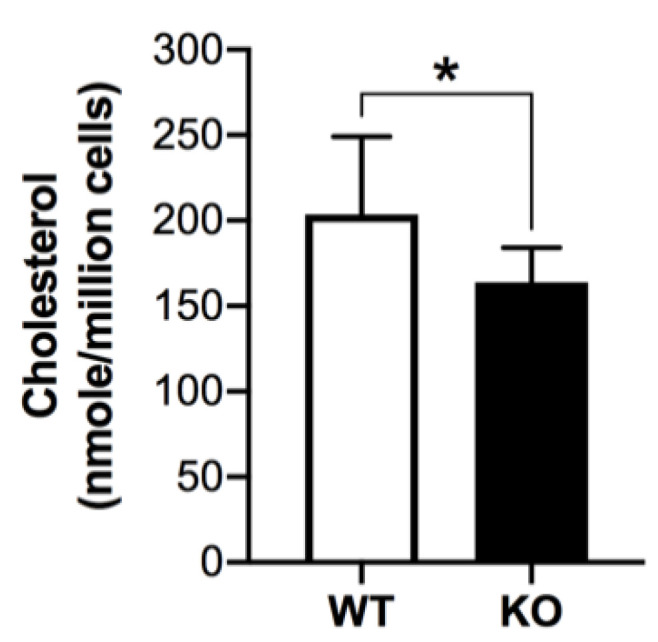
Decreased amounts of total cholesterol in Sertoli cells from 8-month-old *Arsa* KO mice as compared with Sertoli cells from age-matched WT mice. * denotes a significant difference (*p* < 0.05) of the cholesterol amounts between the two samples. Results shown are from six replicate experiments.

The levels of total cholesterol (free + cholesteryl esters), as analyzed by Amplex Red assay with the inclusion of cholesterol esterase in the reaction mixture, were significantly decreased in *Arsa^−/−^* Sertoli cells, as compared with those in WT Sertoli cells (i.e., 164.21 ± 20.20 versus 204.01 ± 45.45 nmole/10^6^ cells, *p* < 0.05) (Figure 4).

ESI-MS/MS revealed that there was a trend towards decreased levels of various molecular species of PCs and SMs in *Arsa^−/−^* Sertoli cells relative to those in WT counterparts. However, except for lysoPC (C16:0), these decreases were not statistically different (Supplemental Figure S3). The levels of PEs and CEs also appeared to be similar in *Arsa^−/−^* and WT Sertoli cells (Supplemental Figures S4 and S5). 

### 3.3. Increased ROS Levels in Sertoli Cells, Testicular Germ Cells, and Seminiferous Tubules of Arsa Null Mice 

**Figure 5:**
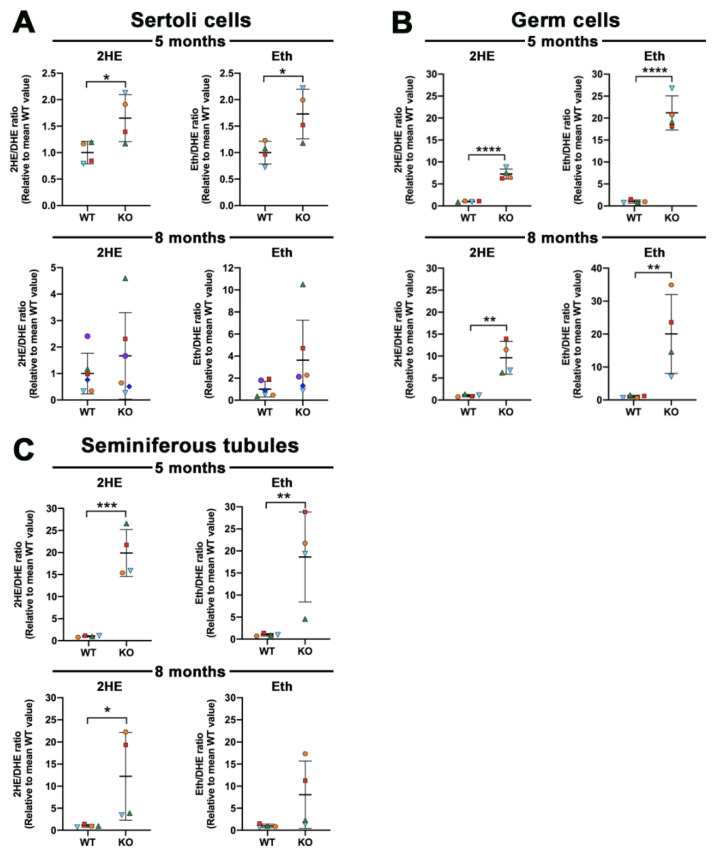
ROS levels in Sertoli cells (**A**), testicular germ cells (**B**) and seminiferous tubules **(C)** from *Arsa* KO mice and age-matched WT mice, 5 months and 8 months of age. Cells and minced tissues were incubated with DHE. Superoxide generated intracellularly converted DHE to 2HE, whereas other ROS and oxidants changed DHE to Eth. Changes of 2HE/DHE and Eth/DHE in cells and tissues from *Arsa* KO mice were expressed as fold increases from corresponding values from age-matched WT mice. Results on Sertoli cells from 8-month-old WT and *Arsa* KO mice were from 6 replicate experiments, whereas those on Sertoli cells from 5-month-old WT and *Arsa* KO mice, and testicular germ cells and seminiferous tubules from WT and KO mice of both ages were each from 4 replicate experiments. *, **, ***, **** denote *p* < 0.05, 0.01, 001 and 0.0001, respectively.

Following incubation with DHE, the levels of 2HE, representing superoxide anion, in Sertoli cells from *Arsa^−/−^* mice were increased as compared with the corresponding levels in Sertoli cells from age-matched WT mice. However, the increase was only significant statistically in Sertoli cells isolated from 5-month-old mice (Figure 5A). A similar result was observed with the levels of Eth (representing other ROS and other oxidants inside the cells) (Figure 5A). Testicular germ cells incubated with DHE also showed increases in both 2HE and Eth in *Arsa^−/−^* mice of both ages, and all increased levels in the *Arsa^−/−^* samples, as compared with the WT samples, were significant (Figure 5B). Since seminiferous tubules comprise TGCs and Sertoli cells, they were subjected to the same analyses. Increased levels of 2HE and Eth were likewise observed in *Arsa^−/−^* samples, with statistical significance for both 2HE and Eth in seminiferous tubules from 5-month-old *Arsa^−/−^* and WT animals. For seminiferous tubules isolated from animals 8 months of age, only the increase in 2HE (superoxide) levels in *Arsa^−/−^* seminiferous tubules was significant (Figure 5C).

**Figure 6:**
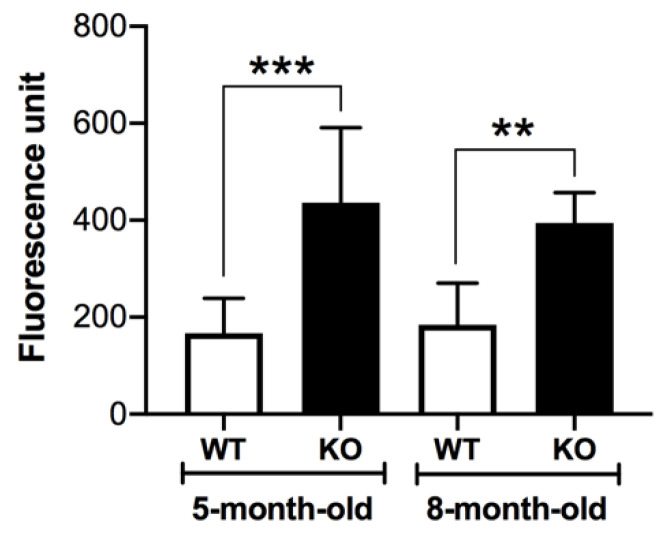
Higher H_2_O_2_ levels in Sertoli cells from 5-month-old and 8-month-old *Arsa* KO mice, as compared with Sertoli cells from age-matched WT counterparts. Results were from 3 replicate experiments. **, *** denote *p* < 0.01 and 0.001, respectively.

Since Sertoli cells are known to possess superoxide dismutase (SOD) [29,30], superoxide anion produced in these cells would continuously be converted to H_2_O_2_, which could be directly quantified by the Amplex Red assay. Figure 6 shows that the levels of H_2_O_2_ in Sertoli cells from both 5-month-old and 8-month-old *Arsa^−/−^* mice were significantly higher than those in Sertoli cells from age-matched WT animals.

### 3.4. Increased Apoptosis in the Primary Cultures of Sertoli cells from 8-Month-Old Arsa^−/−^ Mice

**Figure 7:**
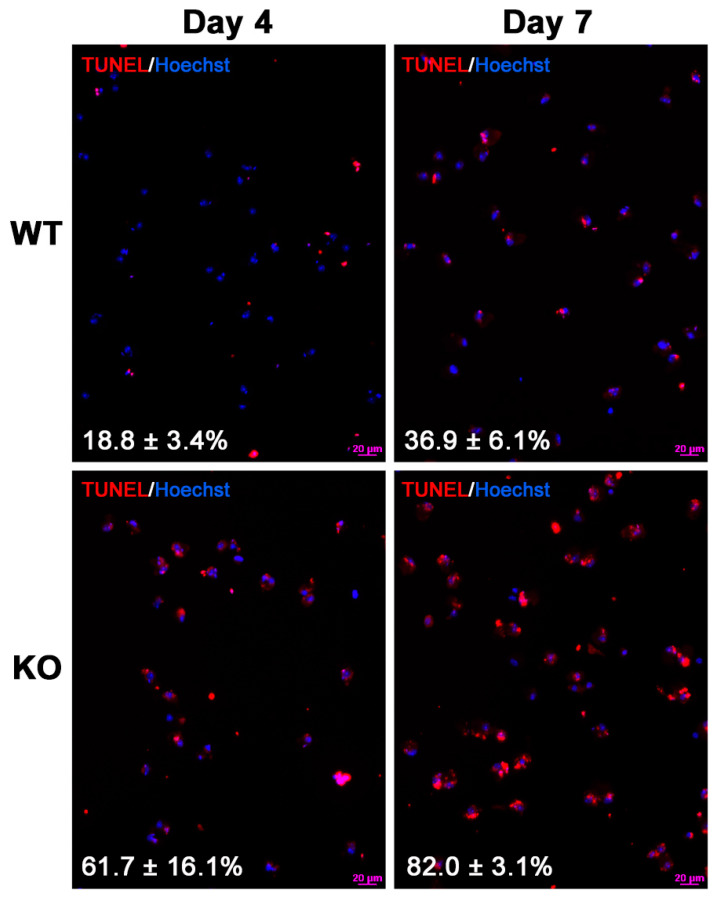
Sertoli cells from 8-month-old *Arsa* KO mice underwent higher rates of apoptosis in culture than Sertoli cells from age-matched WT males. Sertoli cells from *Arsa* KO and WT mice were cultured in parallel and the TUNEL assay was performed on culture day 4 and day 7. Fluorescent images of Sertoli cells from WT and KO mice showing incorporation of TMR-dUTP (red fluorescence) into the nucleus (Hoechst blue fluorescence) were displayed along with percentages of apoptotic cells (TMR-dUTP positive) in the bottom left. Results were from 3 replicate experiments.

**Figure 8:**
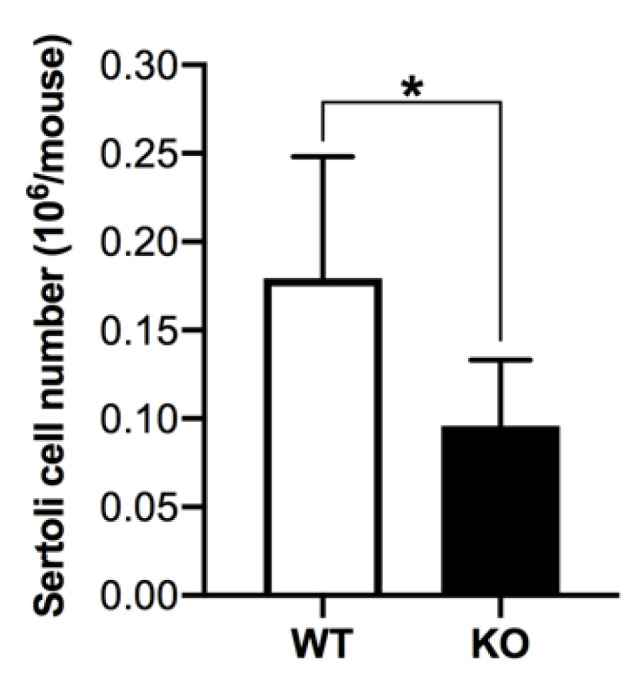
Lower yields of cultured Sertoli cells from 8-month-old *Arsa* KO mice, compared with those from age-matched WT mice. Sertoli cells were detached by Accutase treatment from the culture plate on culture day 7 and counted under a microscope. Results were from 4 replicate Sertoli cell isolations from the WT and KO animals. Four WT mice and 7-8 KO mice were used in each Sertoli cell isolation. * denotes *p* < 0.05.

Spermatogenesis occurs at a 50% wild type rate in 8-month-old *Arsa^−/−^* mice [17]. Considering that Sertoli cells have important roles in supporting TGC development during spermatogenesis [1], we investigated whether Sertoli cells were impaired and/or lower in number in 8-month-old *Arsa^−/−^* mice, as compared with those in age-matched WT mice. Herein, we showed that the primary cultures of Sertoli cells from *Arsa^−/−^* mice at this age had much higher rates of apoptosis as compared with Sertoli cells from counterpart WT mice. On culture day 4, the apoptosis rate was only 18.8 ± 3.4% in the WT Sertoli cell culture but it was 61.7 ± 16.1% in the *Arsa^−/−^* Sertoli cell culture (*p* < 0.0001). On culture day 7, the difference in the apoptotic rates between WT and *Arsa^−/−^* cultures was also significant, i.e., 36.9 ± 6.1% for WT Sertoli cells and 82.0 ± 3.1% for *Arsa^−/−^* Sertoli cells (*p* < 0.0001) (Figure 7). When Sertoli cells were isolated from 8-month-old *Arsa^−/−^* mice or counterpart WT animals and plated for attachment to the culture dish surface, the numbers of adherent Sertoli cells per mouse from *Arsa^−/−^* mice were only half of the values from WT animals (Figure 8). This increased rate of apoptosis in *Arsa^−/−^* Sertoli cells may have occurred in vivo. As expected, the numbers of TGCs isolated from each 8-month-old *Arsa^−/−^* mouse were also 50% of the numbers from each age-matched WT mouse (Supplemental Figure S6).

### 3.5. Increased ROS Levels in the Epididymis of Aging Arsa Null Mice

**Figure 9:**
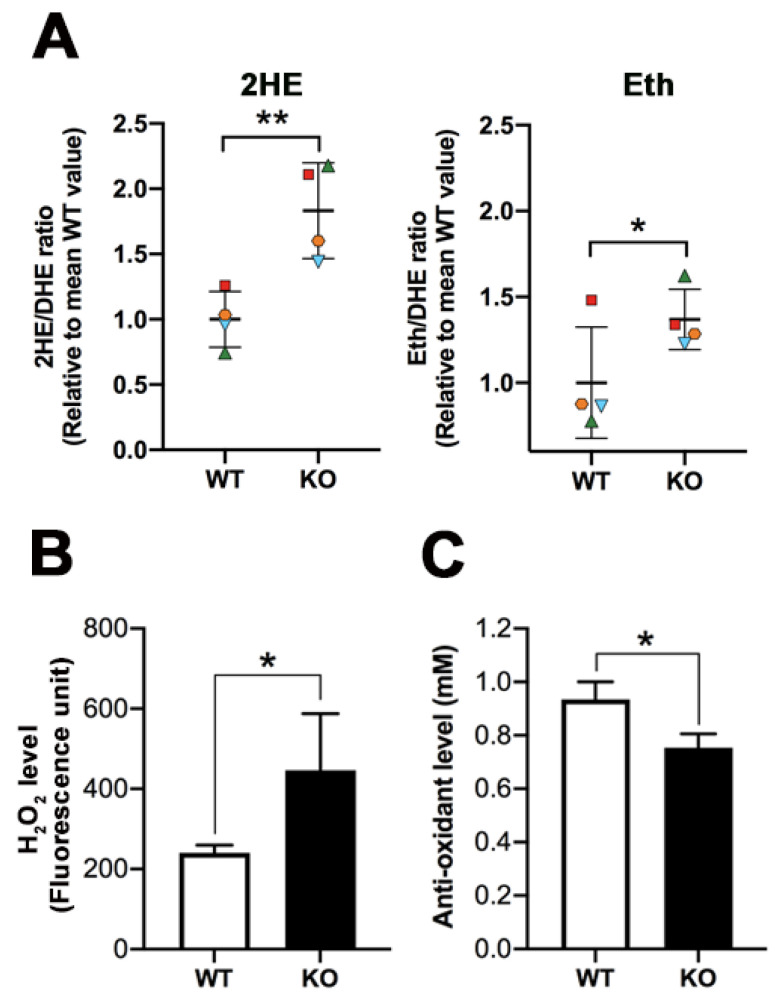
(**A**)**.** Higher superoxide anion levels (2HE) and other ROS plus oxidants (Eth) in the cauda epididymis of 8-month-old *Arsa* null mice, compared with age-matched WT mice. Data from the KO mice were expressed as fold increases over the corresponding values from WT mice in a similar manner as described in the Figure 5 legend. Results were from four replicate experiments. Higher amounts of H_2_O_2_ (**B**) but lower levels of antioxidants (**C**) in the caudal epididymal fluid of 8-month-old *Arsa* KO mice, compared with the counterpart fluid of age-matched WT mice were also displayed. Results on both quantifications were from three replicate experiments. *, ** denotes *p* < 0.05 and 0.001, respectively.

Significant increases in 2HE (superoxide) and Eth (other ROS and oxidant) levels were observed in the cauda epididymis from 8-month-old *Arsa^−/−^* mice, as compared with age-matched WT mice (Figure 9A). Similar results were also noted in the cauda epididymis of 5-month-old animals, as well as in the caput epididymis of both 5-month-old and 8-month-old mice (Supplemental Figure S7). The levels of H_2_O_2_ in the caudal epididymal fluid were also higher in *Arsa^−/−^* mice relative to the WT counterparts (Figure 9B). The lower levels of antioxidants, as measured from the caudal epididymal fluid, in *Arsa^−/−^* mice (Figure 9C), could partially contribute to the higher levels of ROS in this tissue of the KO mice.

### 3.6. Aberrant Levels of Sulfolipids and Abnormal Morphology of Sperm from Aging Arsa^−/−^ Mice

**Figure 10:**
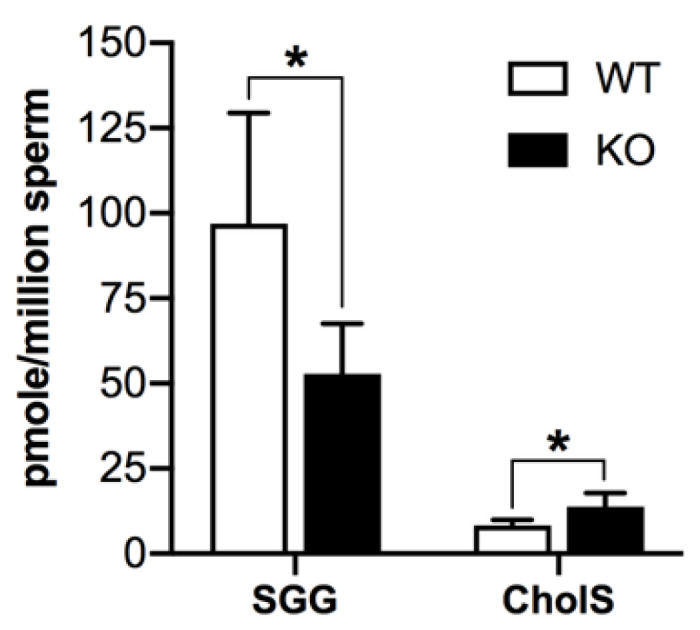
Changes of sulfolipid levels in sperm from 8-month-old *Arsa* KO mice, as compared with sperm from age-matched WT mice. Two main sperm sulfolipids, SGG and cholesterol sulfate (CholS) were quantified by ESI-MS/MS-MRM. Results were from three replicate analyses. In each analysis, 3 and 10 WT and *Arsa* KO mice were used to prepare Percoll-gradient-centrifuged sperm, respectively, from which lipids were extracted for lipidomics work. * denotes *p* < 0.05.

Our previous studies indicate that caudal epididymal sperm from 8-month-old *Arsa^−/−^* mice have minimal fertilizing ability, and our SGG quantification in pooled sperm samples also reveal a 50% level in the KO mice, as compared with age-matched WT animals [17]. In this report, our sulfate precursor ion scanning indicated that SGG and cholesterol sulfate were the two main sperm sulfolipids (Supplemental Figure S8). Quantification of these two sulfolipids from PGC sperm collected separately from *Arsa^−/−^* mice and WT mice, both 8 months of age, revealed that sperm SGG levels in the KO mice were decreased ~50%, as compared with the corresponding WT levels (Figure 10), a result corroborating our previous observation [17]. In contrast, the levels of sperm cholesterol sulfate in *Arsa^−/−^* mice increased to about twice the amounts in WT mice (Figure 10).

**Figure 11:**
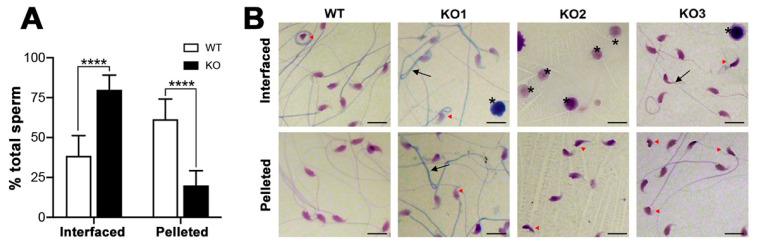
An increase in sperm with abnormal morphology in *Arsa* KO mice, 8 months of age, as compared with age-matched WT sperm. Caudal epididymal sperm from *Arsa* KO and WT mice were separately subjected to Percoll gradient (35/70%) centrifugation. (**A)** Distribution of WT and *Arsa* KO sperm interfaced between the two Percoll solutions and those pelleted after Percoll gradient centrifugation. Results were expressed as means ± SDs of total sperm from 7 each of WT or *Arsa* KO sperm preparations, performed in parallel. Sperm (and immature germ cells) retrieved from 2–4 WT or 4–8 *Arsa* KO mice were used in each Percoll gradient centrifugation. **** denotes *p* < 0.0001. (**B**) Select morphological images of sperm sedimented as the pellet or at the Percoll gradient interface. Images from only 1 WT sample were shown as the results were very consistent throughout all WT samples. Images of pelleted and interfaced sperm in three preparations (KO1, KO2 and KO3) from *Arsa* KO mice were shown in comparison. Red arrow heads and black arrows denote abnormality in sperm heads and tails, respectively. Stars indicate the presence of immature germ at the gradient interface cells in the KO samples.

Changes of sperm morphology from the WT characteristics appeared to correlate with aberrant levels of sperm sulfolipids in *Arsa^−/−^* mice. In normal fertile animals, the subjection of caudal epididymal sperm to Percoll density gradient centrifugation results in the separation of sperm into two populations, i.e., pelleted sperm in the bottom of the tube and sperm interfaced between the two Percoll gradient solutions (35/70% gradient or 45/90% gradient). The pelleted sperm population, constituting about 65% of total sperm, has a high fertilizing ability due to normal sperm morphology, and great motility upon resuspension in medium. On the other hand, the interfaced sperm population (the remaining 35%) is mainly immotile with minimal fertilizing ability. Electron microscopic analyses also reveal the abnormal ultrastructure of interfaced sperm, for example, membranated vesicles surrounding the sperm head and midpiece, and incomplete chromatin condensation [20]. The distribution of the pelleted and interfaced sperm populations of 8-month-old WT mice was as expected for normal fertile animals, i.e., 65% and 35%, respectively (Figure 11A). Although the morphology of almost all of the WT interfaced and pelleted sperm was not distinguishable from each other by light microscopy (Figure 11B), the motility of the two sperm populations was drastically different. More than 90% of WT pelleted sperm were motile, whereas the majority of WT interfaced sperm were immotile (data not shown).

The distribution between pelleted and interfaced sperm after Percoll gradient centrifugation from 8-month-old *Arsa^−/−^* mice was 20% and 80% of total sperm, respectively, values significantly different from those of the WT counterparts (Figure 11A). Although the majority of pelleted sperm from *Arsa^−/−^* mice were motile, their progressive velocity was much reduced (~50% of the WT counterparts). Light microscopic analyses also revealed abnormal morphology of these sperm heads (red arrow head) including misshapen heads, heads of reduced sizes and heads detached from tails. Sperm tails showing 180° folding towards the heads (black arrow) were also observed (Figure 11B). In the interfaced fraction, a higher number of sperm with the same abnormal morphology were observed. In addition, a number of immature round germ cells were present in the interfaced fraction. The percentages of these round germ cells (star) were variable and could be up to 60%, such as that observed in *Arsa* KO mouse #2 (Figure 11B).

## 4. Discussion

In this report, we demonstrated that SGG was co-localized with ARSA and the coenzyme, saposin B, in the lysosomes in the primary Sertoli cell cultures of 8-month-old WT animals (Figure 1 and Figure 2). However, in Sertoli cells of age-matched *Arsa* null mice, SGG was accumulated not only in the distended lysosomes but also in the cytoplasm and the plasma membrane (Figure 2). The marked increase in SGG amounts in these *Arsa* null Sertoli cells was demonstrated by both immunofluorescence and quantitative ESI-MS/MS (Figure 2 and Figure 3). Since Sertoli cells do not have enzymes in the biosynthesis pathway of SGG [1], the results indicated that the accumulated intracellular SGG were from apoptotic TGCs and residual bodies, which were phagocytosed by Sertoli cells. The accumulation of SGG together with the distention of lysosomes in *Arsa^−/−^* Sertoli cells is a typical feature of LSD, which can result in a number of pathogenic cascades [18]. The increase in oxidative stress has been described in other LSDs (e.g., Fabry, Gaucher, Nieman–Pick C1/C2 diseases) [31,32,33,34]. Although ROS at low amounts is beneficial for various physiological processes including the support of spermatogenesis [35,36], ROS at high concentrations in the oxidative stress state will lead to oxidative damages to DNA, proteins and lipids [37,38,39]. With the apparent impairment of *Arsa^−/−^* Sertoli cells ([17]; Figure 7 and Figure 8), we therefore first determined relative levels of superoxide anion and H_2_O_2_ in these cells. Our results indeed indicated increased levels of superoxide anion and H_2_O_2_ in *Arsa^−/−^* Sertoli cells from mice both 5 months and 8 months of age, as compared with WT Sertoli cells from age-matched mice (Figure 5 and Figure 6). Our results corroborate the previous findings describing increased levels of ROS in induced pluripotent stem cells (iPSCs) generated from MLD patients (having *Arsa* mutations and the intracellular accumulation of sulfatides (SGG’s analogues)) as well as their derived cells. Notably, all of these cells exhibit lysosomal distention due to the sulfatide accumulation [40], a result analogous to our observation in Sertoli cells of aging *Arsa* null Sertoli cells. 

The production of superoxide anion occurs mainly during the mitochondrial electron transport chain [41,42]. Since lysosomes and mitochondria are closely associated with each other both physically and functionally, with cross-talk communication between the two organelles [43], the distention of lysosomes with aberrant activity in processing sulfated glycoconjugates in *Arsa* null Sertoli cells may cause their mitochondria to produce increased amounts of superoxide, as compared with WT Sertoli cells. In fact, the superoxide production was statistically higher in *Arsa^−/−^* Sertoli cells than in WT counterparts in 5-month-old animals. However, the corresponding comparison in 8-month-old *Arsa^−/−^* and WT animals only showed a trend, but without a significant difference (Figure 5A). This disparity may be from substantial increases in ROS production due to aging [44], occurring in both WT and *Arsa* null mice. Specifically, the 2HE/DHE amount (reflecting superoxide level) in Sertoli cells from 8-month-old WT mice was 200 nmole/μmole, 3.6 times of the corresponding value from 5-month-old WT animals (i.e., 55 nmole/μmole). This large increase owing to aging would diminish the sharp difference in the superoxide amounts in Sertoli cells, attributed to Sertoli lysosomal swelling in aging *Arsa^−/−^* mice. 

Sertoli cells possess SOD, which generates H_2_O_2_ from superoxide [29,30]. H_2_O_2_ can be further processed into non-reactive products (e.g., water) by scavenger enzymes such as catalase and glutathione-dependent enzymes [29,30,45,46]. Notably, the levels of H_2_O_2_ in Sertoli cells from WT males both 5 months and 8 months of age were comparable (Figure 6), indicating that the increased amounts of superoxide due to aging could be effectively processed and WT Sertoli cells at both ages could survive and function at these baseline H_2_O_2_ amounts. Sertoli cells from 8-month-old WT mice are morphologically the same as those from younger males (our unpublished observations). In contrast, the levels of H_2_O_2_ in Sertoli cells from *Arsa* KO males were significantly higher than those in Sertoli cells from age-matched WT males at both 5 months and 8 months of age (Figure 6). These results suggest that the ROS scavenger enzymes of Sertoli cells in *Arsa* KO mice may not function well. It was possible that part of H_2_O_2_ produced in *Arsa* null Sertoli cells may have been converted to OH^⋅^ radical via the Fenton reaction, catalyzed by Fe^2+^ [39,47]. The highly reactive OH^⋅^ would react instantaneously and irreversibly with biomolecules including DNA, proteins (including ROS scavenger enzymes) and lipids [37,39]. In particular, its reaction with polyunsaturated fatty acids (PUFA, known to be present in Sertoli cells [48,49]) would lead to lipid peroxidation and formation of lipid aldehydes, which would further form adducts with DNA and proteins [50], including those involved in the mitochondrial electron transport chain. The latter would result in further increase in ROS production [51]. These OH^∙^-induced molecular damages would accrue with time, and this may be the cause that Sertoli cells from 8-month-old *Arsa* null mice became smaller with nuclei dislocated towards the basement membrane, while Sertoli cells from 5-month-old KO males were still morphologically normal [17]. The high apoptosis rate of Sertoli cells from 8-month-old *Arsa* null males (Figure 7) may also reflect the oxidative damages occurring in vivo.

In the male reproductive system, ARSA activity is the highest in Sertoli cells [1]. Therefore, the increased ROS levels in Sertoli cells of *Arsa* null mice were not unexpected. In contrast, the finding that superoxide levels were also elevated in TGCs of *Arsa* KO mice (Figure 5B) needs an explanation on its mechanism. Since H_2_O_2_ can permeate the cell membranes [52], it could be speculated that a portion of newly formed H_2_O_2_ could exit Sertoli cells into the adluminal compartment and then enter developing TGCs residing in this space. Due to higher levels of H_2_O_2_ produced by *Arsa* null Sertoli cells, the entry of H_2_O_2_ into TGCs would be at higher rates in the KO animals. Formation of the OH^∙^ radical may then occur with a consequence of molecular and then cellular damages in an analogous manner to that postulated for Sertoli cells. In particular, TGCs are very enriched in PUFA-containing lipids [53,54], and therefore, lipid aldehydes could be formed following the reaction of these lipids with OH^∙^. As described for Sertoli cells above, this could also lead to the increased production of superoxide by mitochondria of *Arsa* null TGCs, as observed in our study (Figure 5). Accumulated molecular damages due to oxidative stress would lead to cellular impairment in due time, as evidenced by a high apoptosis rate of TGCs as well as the dislodgement of some TGCs from the seminiferous tubules into the epididymal lumen in 8-month-old *Arsa* KO mice [17]. The presence of TGCs in the epididymal lumen could have been one cause of higher levels of superoxide in the epididymis and these TGCs could have also released H_2_O_2_ into the surrounding area, resulting in higher amounts of this ROS in the epididymal lumen of aging KO mice (Figure 9A,B). While the majority of TGCs in aging *Arsa* KO males was still present in the seminiferous tubule epithelium, oxidative damages on their molecular components may have slowed down and/or induced aberrancy in their development into testicular sperm. Additional oxidative damages may have occurred during epididymal maturation of the sperm of aging *Arsa* KO mice because of higher levels of H_2_O_2_ but decreased levels of antioxidants in the epididymal lumen in these mice (Figure 9 B,C). Consequently, the decreases in the spermatogenesis rate (Supplemental Figure S6, [17]) and the increased numbers of sperm with abnormal morphology and immotility (Figure 11) were evident in aging *Arsa* null mice. 

The intracellular accumulation of SGG in Sertoli cells of aging *Arsa* KO mice was associated not only with increased levels of ROS but also a decrease in cholesterol content in these cells (Figure 4). In Sertoli cells of WT mice, SGG existed mainly in the lysosome ready to be catabolized. In contrast, SGG was present in the lysosome as well as in the cytoplasmic vesicles and plasma membrane of Sertoli cells of aging *Arsa* KO mice (Figure 2). Since SGG is the lipid that endows rigidity to biomembranes [55], its presence on the plasma membrane of *Arsa* null Sertoli cells would have made this cell surface aberrantly ordered and perhaps physiologically unfavorable. Likewise, cholesterol is known to provide orderedness to biomembranes [56]. Therefore, a release of cholesterol from the plasma membrane, resulting in the decrease of cholesterol content, would be one avenue in adjusting the biophysical behavior of the *Arsa* null Sertoli plasma membrane towards normalcy. 

In contrast to the SGG accumulation observed in Sertoli cells of aging *Arsa* KO mice, SGG amounts in sperm (Figure 10) and TGCs [17] in these mice were only 50% of the WT values. We have previously explained that this reduction is attributed to decreased activity of the enzymes, CGT and CST, in the biosynthesis pathway of SGG in TGCs [17], and our results herein revealing increases in superoxide anion and thus oxidative stress in these cells supported this postulation. The marked decrease in SGG levels would have caused reduced stability to the plasma membrane of *Arsa* null sperm. However, the levels of cholesterol sulfate, which could be synthesized in the epididymis [57,58], were significantly increased in *Arsa* null sperm (Figure 10). Since cholesterol sulfate [57] is known as a sperm plasma membrane stabilizer, its increase would allow the *Arsa* null sperm plasma membrane to regain its orderedness at least partly.

In summary, our studies herein demonstrated that the intracellular accumulation of SGG in Sertoli cells in *Arsa^−/−^* was likely the main cause of increased ROS production in these cells as well as TGCs, and subsequently the prolonged oxidative damages led to observed dysfunctions of Sertoli cells and subfertility in aging *Arsa* null mice [17]. Our study can serve as a model for an investigation of how oxidative stress of Sertoli cells can result in male infertility/subfertility.

## 5. Conclusions

Using *Arsa* null male mice as an experimental model, we reported herein that accumulation of SGG (seminolipid) in ARSA deficient Sertoli cells led to increases in ROS levels, and then molecular and cellular damages. The oxidative stress in these reproductive cells likely was the cause of the subsequent increases in ROS in TGCs and thus a decreased rate of spermatogenesis as well as formation of sperm with abnormal morphology and physiology.

## Data Availability

All data from this study are contained within this article and supplemental information.

## Supplementary Materials

Table S1 and Figures S1–S8 as well as their legends are available online at figshare repository: https://doi.org/10.6084/m9.figshare.13697122.v1.
